# Ferroptosis in Ovarian Cancer: A Novel Therapeutic Strategy

**DOI:** 10.3389/fonc.2021.665945

**Published:** 2021-04-29

**Authors:** Lanyu Li, Cheng Qiu, Min Hou, Xinyu Wang, Changzhen Huang, Jialin Zou, Tianyi Liu, Jinfeng Qu

**Affiliations:** ^1^ Department of Obstetrics and Gynecology, Jinan Central Hospital, Cheeloo College of Medicine, Shandong University, Jinan, China; ^2^ Cheeloo College of Medicine, Shandong University, Jinan, China; ^3^ Department of Orthopaedic Surgery, Qilu Hospital, Cheeloo College of Medicine, Shandong University, Jinan, China; ^4^ Department of Obstetrics and Gynecology, Qilu Hospital, Cheeloo College of Medicine, Shandong University, Jinan, China

**Keywords:** ferroptosis, ovarian cancer, lipid metabolism, reactive oxygen species (ROS), immunotherapy

## Abstract

Ovarian cancer (OVCA) is one of the most lethal malignancies with a five-year relative survival below 50% by virtue of its high recurrence rate and inadequate early detection methods. For OVCA patients, modern approaches include debulking surgery, chemotherapies, angiogenesis inhibitors, poly ADP-ribose polymerase (PARP) inhibitors, and immunotherapies depending on the histological type and staging of the tumor. However, in most cases, simple standard treatment is not satisfactory. Thus, a more effective way of treatment is needed. Ferroptosis is a newly recognized type of regulated cell death marked by lipid peroxidation, iron accumulation and glutathione deprivation, having a connection with a variety of disorders and showing great potential in anti-tumor therapy. Intriguingly, a possible connection between ferroptosis and OVCA is shown on the basis of previously published findings. Furthermore, a growing number of ferroptosis protection pathways have been identified during the past few years with increasing ferroptosis regulators being discovered. In this review, we summarized several major pathways involved in ferroptosis and the study foundation of ferroptosis and ovarian cancer, hoping to provide clues regarding OVCA treatment. And some important issues were also raised to point out future research directions.

## Introduction

Ovarian cancer (OVCA), which frequently manifests as abdominal distension, abdominal or pelvic bloating and abdominal mass at advanced stages (1), is one of the most lethal malignancies by virtue of its high recurrence rate and inadequate early detection methods (2), placing a heavy burden on patients and the society. Histologically, OVCA contains a wide range of tumors, including those of epithelial, sex cord-stromal and germ cell origin, among which, epithelial ovarian cancer is the most common type. Although a significant decline in the incidence and mortality of ovarian cancer has been witnessed during the past few decades due to the improvement of treatment, OVCA still possesses a high mortality rate with a five-year relative survival below 50% (3). It was reported that there were approximately 313,959 new OVCA cases in 185 countries in 2020 with 207,252 new OVCA deaths, accounting for 1.6% and 2.1% of all new cancer cases and new cancer deaths, respectively (4). Furthermore, great efforts have been made to uncover the potent therapeutic strategies and the pathogenesis of OVCA, but the mystery of OVCA is not yet unraveled and still catches many researchers’ eyes.

Modern approaches to patients with OVCA vary from person to person according to the histological type and staging of the tumor, comprising debulking surgery, chemotherapies containing platinum and docetaxel etc., angiogenesis inhibitors, poly ADP-ribose polymerase (PARP) inhibitors, and immunotherapies. Generally speaking, standard treatment for OVCA involves debulking surgery followed by combination chemotherapies based on platinum (5). However, for some patients, especially the ones with recurrent diseases, simple standard treatment is not satisfactory in most cases.

Tumor angiogenesis, which is the formation of novel blood vessels in tumor entities to supply oxygen and nutrients, greatly contributes to tumor growth, progression and metastasis (6). It has been validated that angiogenesis occurs in many cancerous disease contexts, including OVCA; thus, angiogenesis inhibitors, such as bevacizumab should be considered to treat ovarian cancer (7). On the other hand, the detrimental somatic mutation of BRCA genes, which mainly function as a protector in homologous recombination DNA repair pathway, is another frequent molecular event that happens to patients with OVCA (8, 9). Consequently, drugs developed for targeting DNA damage, PARP inhibitors for example, could be employed to treat OVCA patients with such gene mutations. Interest in the relationship between immunotherapies and OVCA is burgeoning with many immune checkpoint pathways being discovered, but there is still uncertainty in view of the poorly-understood mechanisms in this malignancy (10).

## ROS and LIPID Peroxidation In OVCA

**Figure 1:**
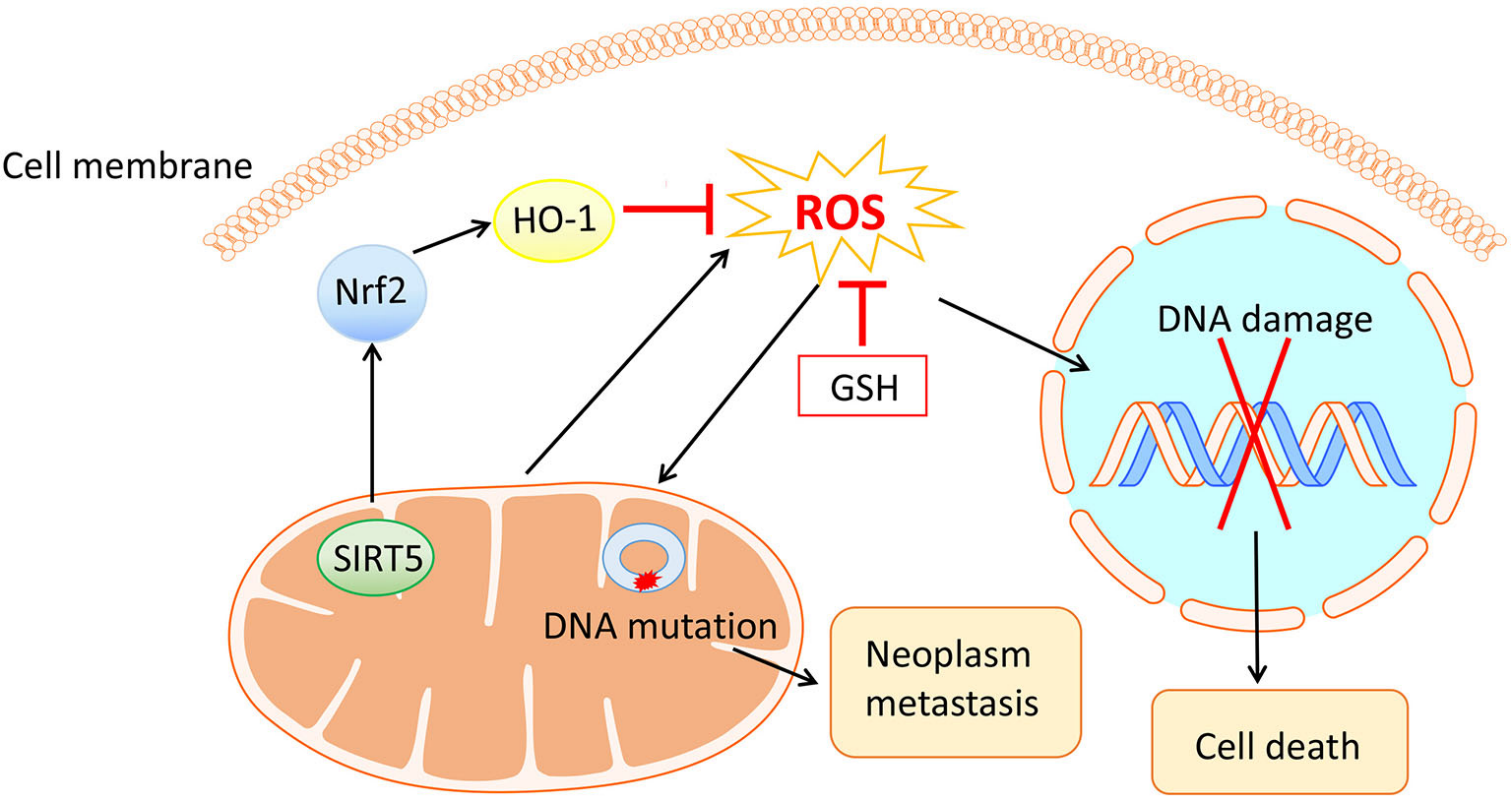
The mitochondria generated ROS and their contradictory effects in ovarian cancer cells. On the one hand, ROS cause DNA damage and thus lead to cell death. On the other hand, ROS bring about mitochondrial DNA mutation, encouraging neoplasm metastasis. Antioxidative systems including GSH and Nrf2 with their upstream and downstream molecules have antagonistic effects on ROS.

Reactive oxygen species (ROS), a group of unstable molecules generated by mitochondria through highly reactive electron transport chain of the mitochondrial respiratory chain (11), commonly consist of singlet oxygen, hydrogen peroxide (H_2_O_2_), and hydroxyl radical, etc. ROS have been reported to participate in various physiological or pathological processes, where metabolism, inflammation and carcinogenesis are involved (12, 13). Besides, an elevated level of ROS that could be eliminated by antioxidative systems covering glutathione (GSH) and nuclear factor erythroid 2-related factor 2 (Nrf2) (14), is observed in cancer cells compared with that of normal cells. Intriguingly, mitochondrial ROS can cause DNA damage and lead to cell death through activating the mitochondrial permeability transition pore pathway (15) whereas a high level of ROS can also result in mitochondrial DNA mutations, giving rise to neoplasm metastasis (16). With the contradictory effects of ROS, anticancer therapies concerning this field should be carefully designed (**Figure 1**).

It has been confirmed that the level of ROS was uplifted in a concentration- and time-dependent manner in several types of OVCA cell lines. A recent experiment conducted by Sun X et al. demonstrated that SIRT5 could suppress ROS by positively regulating Nrf2/heme oxygenase 1 signaling pathway, promoting cell proliferation and cisplatin resistance in OVCA (17) (**Figure 1**). Therefore, these observations suggest that focusing on ROS is an effectual means of coping with OVCA.

Moreover, considerable kinds of lipids are sensitive to ROS-induced oxidation. Under normal circumstances, a homeostasis is maintained between the production and removal of ROS. However, when the homeostasis is impaired with ROS molecules accumulating, the lipid peroxidation process is likely to take place *via* enzymatic or non-enzymatic processes (18, 19). The first step of lipid peroxidation includes the abstraction of a hydrogen atom from the methylene carbon of a fatty acid side chain, mostly polyunsaturated fatty acids by ROS molecules (20). The crushing level of ROS interacts with biological membranes by way of lipid peroxidation, changing the membrane fluidity and permeability; hence the structure and function of a cell are altered (21). Numerous studies have identified the crucial role of lipid peroxidation in ovarian cancer, which provides an emerging strategy of OVCA therapy for us (22, 23).

## Immunotherapy in the Management of OVCA

In the past dozen years, immunotherapies especially immune checkpoint blockade treatment have been widely applied in treating cancers, including non-small cell lung cancer (24) and melanoma (25). Nevertheless, the promising immunotherapy in managing OVCA is still not well understood. It is noteworthy that a destructive tumor microenvironment exists in OVCA patients where there are insufficient T cells, immune suppressive networks and impaired capacity to recognize tumor antigens (10). Hereof, many strategies have been brought forth to regulate the immune system in cancer patients which include immune checkpoint inhibitors targeting programmed death-1 (PD-1)/programmed death ligand-1 (PD-L1) pathway to reinstate an antitumor response, adoptive T cell therapy as well as cancer vaccines etc. Interestingly enough, there was an exhilarating connection between cancer immunotherapy and ferroptosis, as suggested by Wang W et al., and could be manipulated by CD8^+^ T cells (26). Furthermore, Jiang Q et al. discovered that sulfasalazine (SAS)-loaded mesoporous magnetic nanoparticles (Fe_3_O_4_) and platelet (PLT) membrane camouflage, Fe_3_O_4_-SAS@PLT, was able to elicit ferroptosis in mice metastatic tumor models, which then conspicuously potentiated the efficacy of PD-1 immune checkpoint blockade therapy (27).

## Mechanisms of Ferroptosis: the Three Musketeers

**Figure 2:**
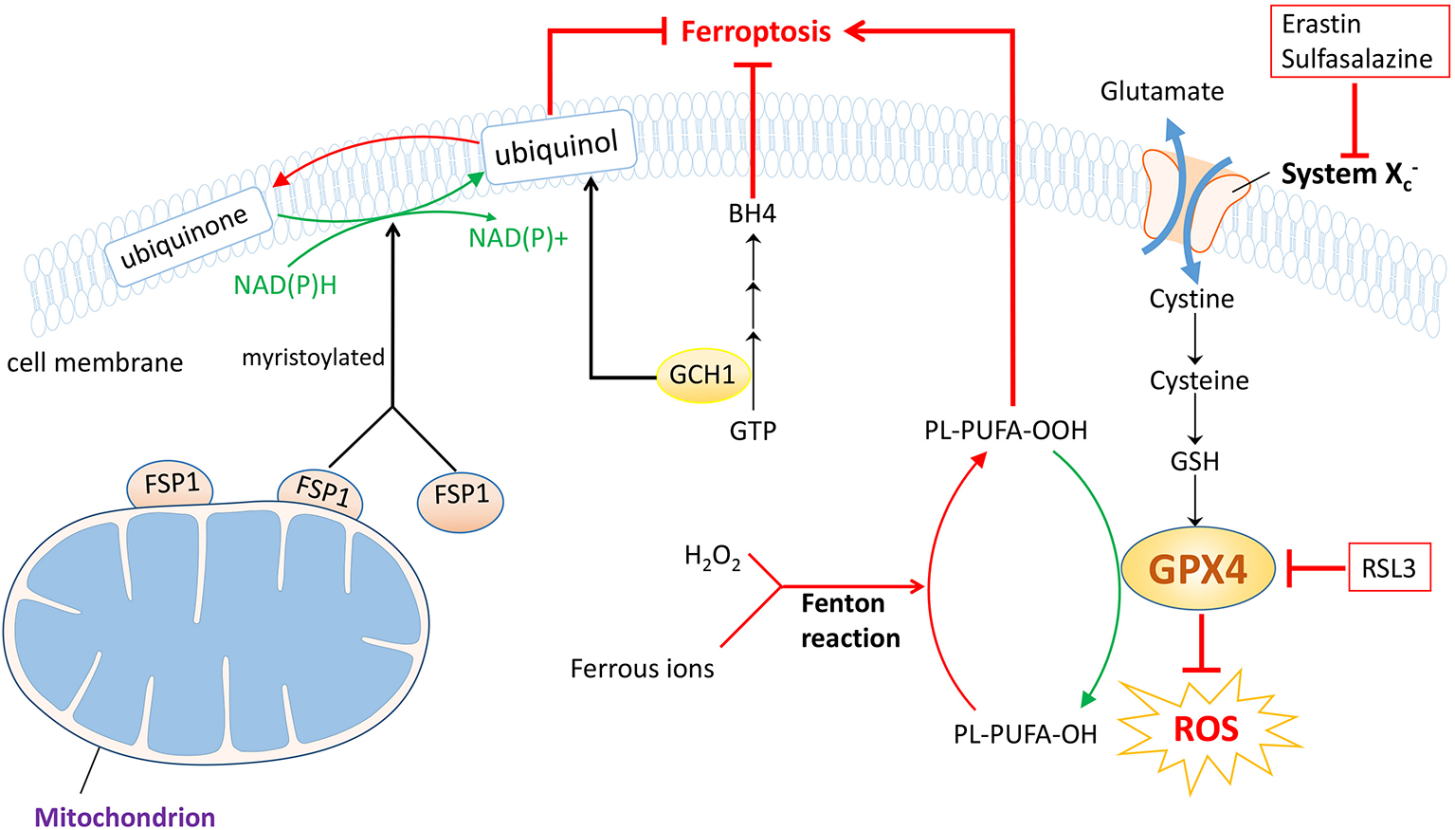
Three prestigious protection pathways implicated in ferroptosis. Of note GPX4 protection pathway is the famous one. With the help of GSH, GPX4 downregulates ROS and inhibits forthcoming ferroptosis, which could be suppressed by RSL3. System $X_{c}^{-}$, the cystine/glutamate antiporter, also functions to synthesize GSH during this process, and can be counteracted by erastin or sulfasalazine. In the FSP1 protection pathway, the myristoylated FSP1 catalyzes the reduction of CoQ10 to ubiquinol, consuming NAD(P)H and mitigating ferroptosis. In the GCH1 protection pathway, GCH1 acts as a rate-limiting enzyme to manage the biosynthesis of BH4, while regulating ubiquinol as well. Therefore, ferroptosis progression is blocked. However, the Fenton reaction, the interaction between ferrous ion and H_2_O_2_, triggers the occurrence of ferroptosis.

Ferroptosis, coined by Dixon et al. was first revealed in 2012, whose hallmarks were lipid peroxidation, iron accumulation and glutathione deprivation (28). Ferroptosis is an important form of regulated cell death, which is biochemically, morphologically and genetically distinctive from a great deal of well-known classes of regulated cell death encompassing apoptosis, necroptosis and autophagy (28). As its name indicates, ferroptosis mediates cell demise in a caspase-independent but iron-dependent way. Hitherto, significant strides have been made to detect the specific mechanisms behind this innovative biological process and the underlying link between ferroptosis and different courses of diseases. Large quantities of evidence signify a close correlation between ferroptosis and a variety of disorders that embrace neurodegenerative diseases (29), ischemia/reperfusion injury (30, 31), acute kidney injury (32), and tumors (33, 34), etc. Additionally, a possible connection between ferroptosis and OVCA is shown on the basis of previously published findings (35, 36), and aiming at ferroptosis may serve as an elaborate scheme to deal with it (37). Herein, we present three predominant pathways that may be closely related to ferroptosis process in ovarian cancer (**Figure 2**).

### GPX4-GSH Protection Pathway

Together with the cystine/glutamate antiporter $\left(system{\text{ X}}_{c}^{-}\right)$, glutathione peroxidase 4 (GPX4)-GSH axis was determined to regulate ferroptotic cancer cell death by Stockwell et al. in 2014 (38), which is now considered as a classical pathway. The duty of system $X_{c}^{-}$, composed of the substrate-specific subunit xCT also known as solute carrier family 7 (SLC7A11) and solute carrier family 3 member 2, is to uptake cystine while evacuating glutamate, which could be inhibited by erastin and sulfasalazine, etc (39). The smuggled cystine is reduced to cysteine and utilized to synthesize GSH, with which acting as a cofactor of the key regulator, GPX4. In coordination with GSH, GPX4 holds the ability to neutralize ROS and defy oxidation and could be choked with the existence of RSL3 (28). As a matter of course, ferroptosis process is suppressed (**Figure 2**). Thereby, agents developed to fight against this axis will trigger ferroptosis and may offer a potent approach to treating ovarian cancer. Also, the exact responsibility of iron nevertheless stays unclear; as is illustrated, the increased level of ferrous ion reacts actively with H_2_O_2_, known as Fenton reaction and eventually the cancer cells undergo ferroptosis (40) (**Figure 2**).

### FSP1-CoQ10 Protection Pathway

Apart from GPX4, another dazzling star ferroptosis-suppressor-protein 1 (FSP1) is rising in the field of ferroptosis. FSP1 which was renamed from apoptosis-inducing factor mitochondrial 2 (AIFM2), was first found to be relevant to ferroptosis in 2019 stated by two back-to-back investigations (41, 42). Since the first discovery of AIFM2, it has been proved to prompt cell death in a caspase-independent way (43–45). Later AIFM2 was detected to abrogate cell death *via* ferroptosis that strikingly resembles that of GPX4; thereafter AIFM2 was changed into FSP1. A majority of FSP1 is attached to outer membrane of mitochondrion while the left is cytosolic protein. So when myristoylated, FSP1 moves to plasma membrane to neutralize ROS, preventing lipid peroxidation and subsequent ferroptosis. FSP1 exerts defensive effects with the help of coenzyme Q10 (CoQ10), also known as ubiquinone, consuming NAD(P)H as well (46). Briefly, ubiquinol is obtained by reduction of ubiquinone catalyzed by FSP1, an NADH-dependent CoQ10 oxidoreductase and the produced ubiquinol ameliorates oxidation through radical trapping. Ultimately, the lipid peroxidation process is blocked and so is the ferroptosis process (**Figure 2**). In short, medications downregulating FSP1 could be exploited to facilitate ferroptosis and become a potentially curative measure to treat ovarian cancer.

### GCH1-BH4 Protection Pathway

Parallel to but independent of GPX4 and FSP1 protection pathways, recent advances highlighted the key role of GTP cyclohydrolase 1 (GCH1)-tetrahydrobiopterin (BH4) pathway in ferroptosis regulation (47). Early reports have verified that GCH1 is a governing rate-limiting enzyme in the synthesis of BH4, derived from GTP (48) and having the outstanding capacity to eliminate lipid peroxidation (47). Afterwards, ferroptosis is arrested. In addition, Kraft et al. ascertained that the generation of BH4 could be enhanced by GCH1 overexpression and that GCH1 overexpression abolished the deleterious effects of RSL3-induced ferroptotic cell death. More importantly, it was also implied that the level of GCH1 expression determined cancer cell resistance to ferroptosis and that GCH1/BH4 enriched reduced CoQ10, making further efforts to mitigate ferroptosis progression (47, 49) (**Figure 2**). Despite many unknown facts, the appearance of GCH1-BH4 axis may accordingly represent a potential chemotherapeutic tactic for ovarian cancer therapy.

## Ferroptosis: a Rising Star With Great Therapeutic Potential in OVCA

It is known that *TP53* is an outstanding tumor suppressor gene with the capacity to induce apoptosis and has a strong connection with ferroptosis as shown by a number of studies (31, 50–52) while FSP1 is viewed as a p53-inducible gene and a downregulated effector downstream of p53 in tumors (43, 53). A recent experiment conducted by Zhang Y et al. confirmed the relationship between ferroptosis and p53 in OVCA (54). The inhibited viability of OVCA cells incubated in superparamagnetic iron oxides (SPIO)-serum is mostly likely to be associated with transfer of iron oxide nanoparticles to mitochondria and then the intracellular iron accumulation emerges. SPIO-serum facilitates the occurrence of ferroptosis with p53 exerting synergistic functions through downregulating ferroptosis-related proteins, SLC7A11 and GPX4 in OVCA cells. As such, a recently published study validated SPIO nanoparticles’ role of ferroptosis induction in OVCA stem cells whereas cellular autophagy is weakened (55). Despite the clarification of relation between GPX4-GSH protection pathway of ferroptosis and p53 in OVCA, the interaction of p53 with other ferroptosis protection pathways and regulators in OVCA still needs to be interpreted to draw a full picture of p53 and ferroptosis network. And the line between ferroptosis and other kinds of cell death and their incentives remain a problem.

What is more, many studies have focused on oxidative stress metabolism with regard to ferroptosis in OVCA cells. For example, mitochondrial alterations, aberrant ROS production and potentially ferroptosis are found to contribute to elevated chemosensitivity in human OVCA (56). In one study, it was found that the survival of ovarian clear cell carcinoma relies on the access to cysteine and that cysteine depletion impairs the cardinal protection pathway, namely the GPX4-GSH pathway of ferroptosis, consequently eliciting oxidative stress-induced ferroptosis concurrent with necrosis, another famous type of cell demise. Furthermore, mitochondrial metabolism is also modified resulting from lack of cysteine and so is the biogenesis of iron-sulfur cluster, making further efforts to mitochondrial damage (57). Divertingly, the continued activation of another notorious anti-oxidative system Nrf2 was observed in ferroptosis-resistant OVCA cells and a further step was made towards cysteine and ferroptosis by Liu N et al. (58). They figured that prolonged treatment of erastin could induce ferroptosis resistance instead of inducing ferroptosis in OVCA cells because of continuous upregulation of Nrf2 together with its downstream effector cystathionine β-synthase, a crucial enzyme for the biosynthesis of cysteine. Based on these two studies, the intricate crosstalk between cysteine and other signals in OVCA is worthy of detection.

Beyond the above stated aspects, ferroptosis is in an intimate relationship with the clinic. It was reported that overexpression of transcriptional coactivator with PDZ-binding motif (TAZ), a sensor of cell density, sensitizes OVCA cells to ferroptosis and that in chemoresistant recurrent OVCA cells, lower level of TAZ decreases OVCA cells’ sensitivity to ferroptosis (35). In platinum-tolerant OVCA cells, one study inspected an expression of the Wnt receptor Frizzled-7 (FZD7), which positively alters glutathione metabolism pathways including GPX4. Posterior to exposure to GPX4 inhibitors, FZD7^+^ platinum-tolerant OVCA cells are more likely to experience ferroptosis, opening new avenues for platinum-tolerant OVCA treatment (59). In OVCA cells resistant to another chemotherapy docetaxel due to ATP binding cassette subfamily B member 1 (ABCB1) overexpression, erastin exhibits great ability to reverse the effect of ABCB1, conferring ferroptosis and enhancing the susceptibility to docetaxel in OVCA, proving the synergistic activity of erastin and docetaxel (60). Other than chemotherapeutic drugs, a lately published article shed light on correlation of ferroptosis inducers and PARP inhibitors in BRCA-proficient OVCA. PARP inhibition expedites ferroptosis *via* hampering SLC7A11 in a p53-dependent manner in OVCA (61). With certain studies centering on common treatment approaches targeting GPX4-GSH protection pathway of ferroptosis, other pathways however, are little studied. Delightingly, ferroptosis-related mRNA and genes have been analyzed and identified as therapeutic targets as well as prognostic indicators respectively, exposing new treatment vulnerabilities and offering promising prognostic indicators in OVCA patients (62, 63).

## Discussion

Taken together, ferroptosis is a notable style of regulated cell death that was recognized lately, with three prodigious protection pathways substantiated in succession. The intensive study of mechanisms underlying ferroptosis is of vital significance in mapping its role in all kinds of related carcinomas with compelling evidence denoting a close association between ovarian cancer and ferroptosis. As we now know that docetaxel and PARP inhibitors synergize with ferroptosis inducers in OVCA, studies with respect to ferroptosis and other classical drugs such as cyclophosphamide and vincristine in OVCA still await further elucidation. In addition, it has been reported that mature drugs including sulfasalazine could induce ferroptosis in cancer entities like breast cancer and head and neck cancer (64, 65), their clinical use in OVCA remains poor. Therefore, the expanding clinical usage of those mature drugs towards other malignancies should be considered. Although steady progress has been achieved in terms of ferroptosis, mechanisms underlying the three predominant protection pathways need to be improved and whether there are some other important mechanisms is yet intangible.

Except for the above mentioned issues, a couple of *in vitro* experiments have been conducted to exhibit extraordinary antitumor effects of ferroptosis in OVCA, but a lack of *in vivo* applicable ferroptosis inducers that could be designed as promising drugs does exist. Subsequently, diverse cancer cells display varied susceptibilities to ferroptosis. So it is inevitable to take this consideration into account before making use of ferroptosis regulators as an OVCA therapeutic method. Another problem is the emergence of ferroptosis resistance in OVCA and how to handle this possible misfortune.

Conclusively, the conspicuous exploration of ferroptosis and its regulators may provide potential breakthrough points on anti-OVCA therapies. With the unprecedentedly prosperous investigations concentrating on ferroptosis, a broad application prospect is worth waiting for.

## Author Contributions

TL and JQ raised the idea for the article and critically revised the manuscript. XW, CH, and JZ performed the literature search and data analysis. TL, LL, CQ, and MH were the major contributors in the drafting of the work. All authors contributed to the article and approved the submitted version.

## Conflict of Interest

The authors declare that the research was conducted in the absence of any commercial or financial relationships that could be construed as a potential conflict of interest.

## Abbreviations

OVCA, ovarian cancer; PARP, poly ADP-ribose polymerase; ROS, reactive oxygen species; H_2_O_2_, hydrogen peroxide; GSH, glutathione; Nrf2, nuclear factor erythroid 2-related factor 2; PD-1, programmed death-1; PD-L1, programmed death ligand-1; SAS: sulfasalazine; PLT, platelet; GPX4, glutathione peroxidase 4; SLC7A11, solute carrier family 7; FSP1, ferroptosis-suppressor-protein 1; AIFM2, apoptosis-inducing factor mitochondrial 2; CoQ10, coenzyme Q10; GCH1, GTP cyclohydrolase 1; BH4, tetrahydrobiopterin; SPIO, superparamagnetic iron oxides; TAZ, transcriptional coactivator with PDZ-binding motif; FZD7, Frizzled-7; ABCB1, ATP binding cassette subfamily B member 1.
